# Downregulation of peroxiredoxin-3 by hydrophobic bile acid induces mitochondrial dysfunction and cellular senescence in human trophoblasts

**DOI:** 10.1038/srep38946

**Published:** 2016-12-13

**Authors:** Wei-Bin Wu, Ramkumar Menon, Yue-Ying Xu, Jiu-Ru Zhao, Yan-Lin Wang, Yuan Liu, Hui-Juan Zhang

**Affiliations:** 1 Departments of Pathology and Bio-Bank, the International Peace Maternity and Child Health Hospital, Shanghai Jiao Tong University School of Medicine, Shanghai, China; 2 Institute of Embryo-Fetal Original Adult Disease Shanghai Key laboratory for Reproductive Medicine, School of Medicine, Shanghai Jiao Tong University, Shanghai, China; 3 Division of Maternal-Fetal Medicine and Perinatal Research, Department of Obstetrics and Gynecology, the University of Texas Medical Branch at Galveston, Galveston, Texas, United States of America; 4 Prenatal Diagnosis Center & Fetal Medicine Unit, the International Peace Maternity and Child Health Hospital, Shanghai Jiao Tong University School of Medicine, Shanghai, China

## Abstract

Intrahepatic cholestasis of pregnancy (ICP) is a pregnancy-specific disorder characterised by raised bile acids in foetal-maternal circulation, which threatens perinatal health. During the progression of ICP, the effect of oxidative stress is underscored. Peroxiredoxin-3 (PRDX3) is a mitochondrial antioxidant enzyme that is crucial to balance intracellular oxidative stress. However, the role of PRDX3 in placental trophoblast cells under ICP is not fully understood. We demonstrated that the level of PRDX3 was downregulated in ICP placentas as well as bile acids–treated trophoblast cells and villous explant *in vitro*. Toxic levels of bile acids and PRDX3 knockdown induced oxidative stress and mitochondrial dysfunction in trophoblast cells. Moreover, silencing of PRDX3 in trophoblast cell line HTR8/SVneo induced growth arrest and cellular senescence via activation of p38-mitogen-activated protein kinase (MAPK) and induction of p21^WAF1/CIP^ and p16^INK4A^. Additionally, enhanced cellular senescence, determined by senescence-associated beta-galactosidase staining, was obviously attenuated by p38-MAPK inhibitor SB203580. Our data determined that exposure to bile acid decreased PRDX3 level in human trophoblasts. PRDX3 protected trophoblast cells against mitochondrial dysfunction and cellular senescence induced by oxidative stress. Our results suggest that decreased PRDX3 by excessive bile acids in trophoblasts plays a critical role in the pathogenesis and progression of ICP.

Intrahepatic cholestasis of pregnancy (ICP) is a pregnancy-associated disorder that mainly develops during the second or third trimester of pregnancy. ICP affects 0.2–2% of all pregnant women worldwide, irrespective of ethnicity and geographical differences^1^,^2^. Clinical features of ICP are pruritus, elevated serum total bile acids (TBAs) and liver transaminases in the mother. However, the major concern of the disease is the increased risk of adverse foetal outcomes, including foetal distress, spontaneous preterm labour and sudden intrauterine death, particularly in those severe ICP cases with TBA levels ≥40 μmol/L^3^,^4^,^5^. The aetiology and mechanism of foetal complications associated with ICP is complex and not fully elucidated. Although it was suggested that TBA level in maternal serum during ICP was correlated with adverse foetal/neonatal outcome^3^,^5^, the concentration of TBA in the foetal compartment is much lower than that in the maternal circulation^6^. As the central regulator of maternal-foetal interaction, placenta plays a key role in maintaining foetal health^7^. Thus, placenta may suffer the major insults from elevated maternal bile acids, which would disrupt placental physiology during ICP. The destruction of the normal function of placenta would greatly threaten foetal health.

There is growing evidence that indicates that bile acids–induced oxidative stress is closely associated with the pathogenesis of ICP^8^,^9^,^10^. Severe oxidative stress is present in both human and mouse placenta during the progression of ICP. The trophoblasts in placenta are vulnerable to cytotoxicity mediated by hydrophobic bile acids, such as deoxycholic and chenodeoxycholic acid. These toxic bile acids induced oxidative stress, which leads to accumulation of reactive oxygen species (ROS) in cytoplasm and mitochondria^11^. In normal pregnancy, there is a balance between ROS production and antioxidant scavenging capacity in placenta^12^. However, animal model studies showed that in obstructive cholestasis during pregnancy (OCP), the balance was disrupted by bile acids–induced placental oxidative damage and an impaired antioxidant system^9^,^13^.

Peroxiredoxin (PRDX) is a ubiquitous family of peroxidase enzymes that play dominant roles in scavenging ROS and protecting against intracellular oxidative stress^14^. PRDX isoforms are highly expressed in human tissues, localised in discrete cellular compartment, and influence various cellular processes^15^,^16^. PRDX3, which is specifically localised to mitochondrial matrix, plays an essential role in maintaining mitochondrial homeostasis. In the adrenal cortex, PRDX3 is regarded as the most important enzyme in eliminating mitochondrial H_2_O_2_^17^.

Interestingly, the observation of frequent stillbirth and enhanced placental oxidative stress in *Prdx3*-deficient mice indicates that *Prdx3* plays a crucial role in placental antioxidant defence^18^,^19^. Our previous study also indicated that lowered expression of *Prdx3* might be correlated with placental oxidative stress in a mouse ICP model^8^. However, it was unclear whether downregulation of PRDX3 played a role in toxic bile acid–induced trophoblast damage. The current study investigated the role of PRDX3 in trophoblasts exposed to bile acids. We confirmed the decreased expression of PRDX3 in ICP placentas and in trophoblasts under hydrophobic bile acids treatment. Moreover, knockdown of PRDX3 in trophoblast cells promoted oxidative stress and mitochondrial dysfunction. We also found that PRDX3 knockdown led to oxidative stress–induced cellular senescence via activating p38 mitogen-activated protein kinase (MAPK) and inducing the expression of the cell cycle inhibitors p21^CIP1/WAF1^ and p16^INK4A^.

## Results

### The expression of PRDX3 was decreased in ICP placenta

**Figure 1:**
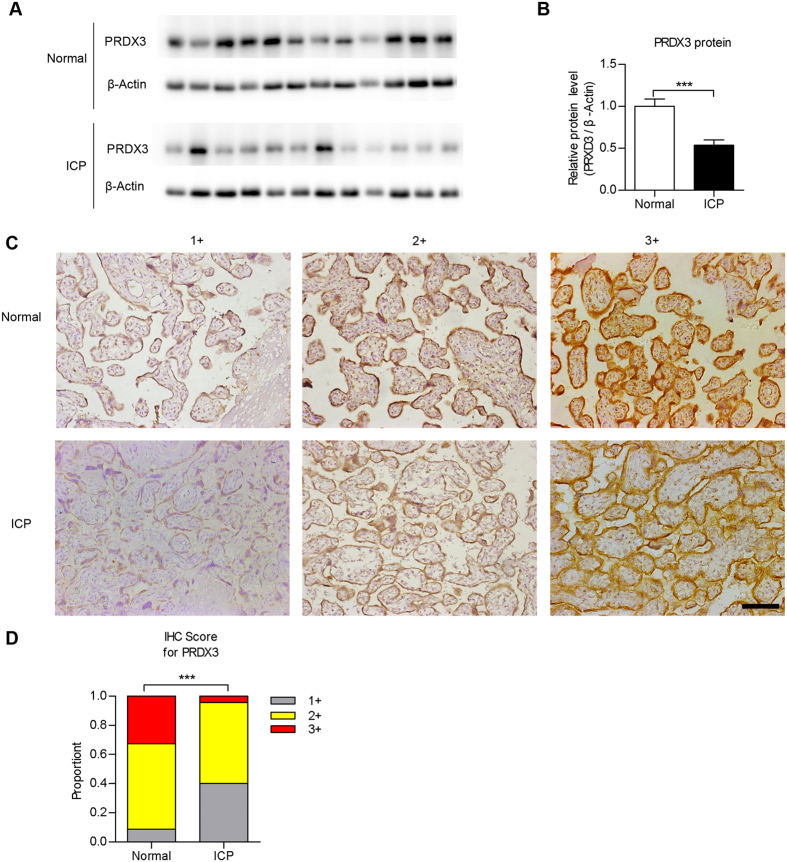
The expression of peroxiredoxin-3 (PRDX3) was decreased in placentas from intrahepatic cholestasis of pregnancy (ICP) patients. (**A**) Immunoblot assay for detecting PRDX3 protein level in placentas from normal pregnancies and ICP patients. Full-length blots were presented in Supplementary Fig. S7. (**B**) Relative protein level of PRDX3 through densitometric analysis of immunoblot results from (**A**) with normalisation to β-Actin (n = 12 in each group, ***p < 0.001, Student’s t test). (**C**) Immunohistochemistry analysis of PRDX3 in trophoblasts of placental tissues. Immunostaining intensity was showed as 1+ (weak), 2+ (moderate) or 3+ (strong). Original magnification 200x, bar: 100 μm. (**D**) Statistically analysis of immunostaining signal of PRDX3 in placentas (n = 46 for normal, n = 70 for ICP, ***p < 0.001, Fisher’s exact test).

First, we examined the expression of PRDX3 in human placental tissues normal pregnancies (control) and ICP patients by immunoblot assay. As shown, the protein level of PRDX3 was significantly lower in ICP placentas compared with those in controls (Fig. 1A,B, n = 12, *p* < 0.001). By immunohistochemistry (IHC) assay, we observed PRDX3 predominantly localised in trophoblastic cells in term placenta. Clinical characteristics of the study population are outlined in Table 1. IHC score was showed as: 1+: weak, 2+: moderate and 3+: strong. In accord with results from immunoblot, a higher percentage of placentas with low PRDX3 level was present in the ICP group than in normal pregnancies (Fig. 1C,D, p < 0.001). Taken together, these findings indicate that the expression of PRDX3 is decreased in trophoblasts of ICP placenta.

### Hydrophobic bile acid instead of promoter hypermethylation contributed to PRDX3 downregulation in trophoblast cells

**Figure 2:**
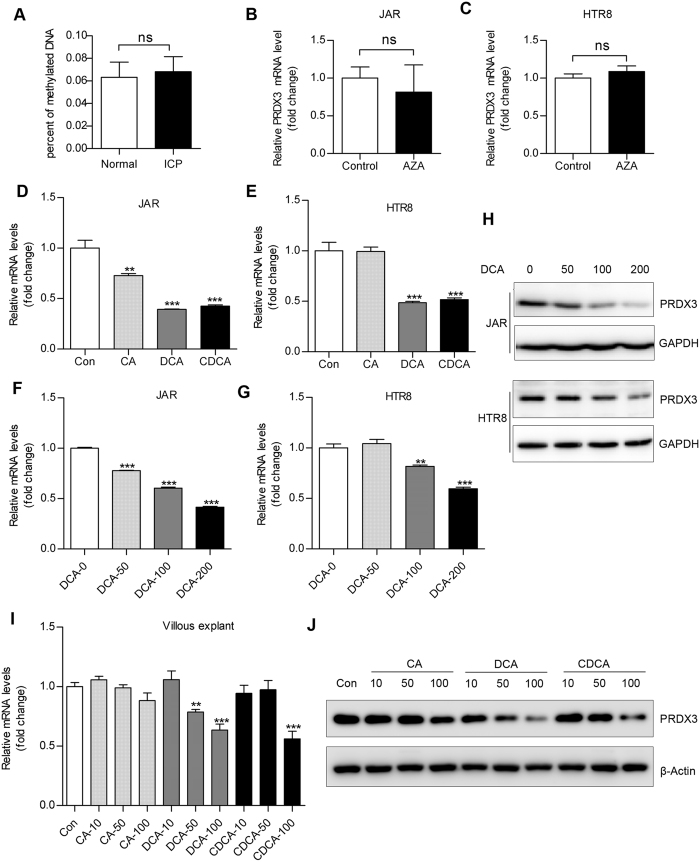
Peroxiredoxin-3 (PRDX3) was downregulated in trophoblastic cells by hydrophobic bile acid instead of promoter hypermethylation. (**A**) DNA methylation analysis for detecting PRDX3 promoter methylation level in placentas from normal pregnancies and intrahepatic cholestasis of pregnancy patients. The percentages of methylated DNA were very low in both groups. JAR (**B**) and HTR8 (**C**) cells were treated with 5 μM 5-aza-2′deoxycytidine (AZA), a specific inhibitor of DNA methylation for 72 hours. After harvest, the messenger RNA (mRNA) level of PRDX3 was determined by quantitative reverse transcription - polymerase chain reaction (qRT-PCR) assay. (**D**,**E**) JAR and HTR8 cells were treated with vehicle (Con), or with μM cholic acid (CA), deoxycholic acid (DCA) and chenodeoxycholic acid (CDCA) for 24 hours. The mRNA level of PRDX3 in JAR (**D**) and HTR8 (**E**) was determined by qRT-PCR assay with normalisation to 18s rRNA level. (**F**,**G**) JAR and HTR8 cells treated with 0, 50, 100 or 200 μM DCA for 24 hours were subjected to qRT-PCR assays for detecting PRDX3 mRNA expression. (**H**) JAR and HTR8 cells were treated with 0, 50, 100 or 200 μM DCA for 24 hours, respectively. After harvest, the protein level of PRDX3 in JAR and HTR8 cell was assayed by Western blot. Glyceraldehyde 3-phosphate dehydrogenase (GAPDH) was used as an endogenous reference. Full-length blots were presented in Supplementary Fig. S8. (**I**,**J**) Villous explants treated with 0, 10, 50 or 100 μM CA/DCA/CDCA for 24 hours were subjected to qRT-PCR assay for detecting PRDX3 mRNA expression (**I**) and Western blot assay for detecting PRDX3 protein level (**J**). Data were shown as mean ± standard error of mean. Statistical significance was determined by Mann-Whitney test, Student’s t test or one-way analysis of variance followed by Dunnett’s post-hoc test. (ns: no significance; *p < 0.05, **p < 0.01, ***p < 0.001, versus the control group).

To determine the possible reasons for reduced PRDX3 expression in ICP placentas, we tested the methylation status of CpG islands within PRDX3 promoter in placental tissues. The PRDX3 promoter hypomethylation was observed in both the normal and ICP groups, with no significant difference (Fig. 2A). Moreover, treatment with DNA methyltransferase inhibitor (5-Aza-2′-deoxycytidine) showed no obvious effect on PRDX3 messenger RNA (mRNA) expression (Fig. 2B,C) in trophoblastic cell lines JAR and HTR8/SVneo (HTR8). On the other hand, administration of hydrophobic bile acids, especially deoxycholic acid (DCA) and chenodeoxycholic acid (CDCA), significantly reduced the mRNA expressions of PRDX3 in JAR and HTR8 cells (Fig. 2D,E). Moreover, treatment with DCA decreased both mRNA (Fig. 2F,G) and protein (Fig. 2H) level of PRDX3 in a dose-dependent manner. Treatment with CA/DCA/CDCA also significantly reduced PRDX3 mRNA and protein level in human villous explants from term placentas (Fig. 2I,J). Taken together, these data indicated that reduced PRDX3 level is not due to promoter hypermethylation, but might be induced by elevated bile acid concentration in ICP placentas.

### Bile acids resulted in mitochondrial dysfunction and trophoblast impairment

**Figure 3:**
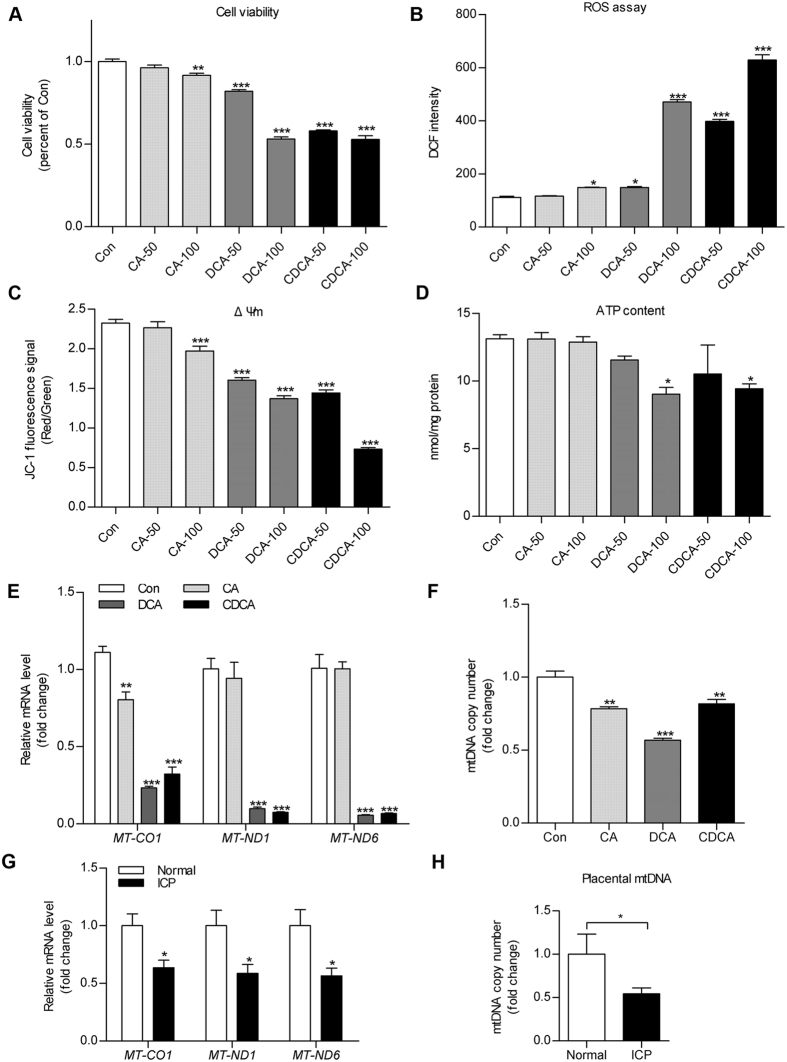
Treatment with bile acids resulted in mitochondrial dysfunction and trophoblast impairment. (**A**–**D**) HTR8 cells were treated with 50 μM or 100 μM cholic acid (CA), deoxycholic acid (DCA) and chenodeoxycholic acid (CDCA) for 24 hours. (**A**) Cell viability, (**B**) ROS production, (**C**) mitochondrial membrane potential (ΔΨm) and (**D**) adenosine triphosphate (ATP) content were assayed using corresponding kits. The ATP level was shown as nmol/mg protein and the others were expressed as arbitrary units. (**E**,**G**) qRT-PCR assay was applied to detect mitochondrial gene transcripts levels (*MT-CO1, MT-ND1* and *MT-ND6* were taken as the representative of mitochondrial genes transcripts) in HTR8 cells treated with 100 μM CA, DCA, CDCA (**E**) and human placental tissues from normal pregnancies or intrahepatic cholestasis of pregnancy patients (**G**). The messenger RNA levels were calculated by normalisation to an internal control 18s rRNA. (**F**,**H**) Mitochondrial DNA copy number was quantified by quantitative polymerase chain reaction assay with normalisation to genomic DNA level in 100 μM CA, DCA and CDCA treated HTR8 cells (**F**) or human placentas (**H**). All experiments were performed in triplicate. Data were shown as mean ± standard error of mean. Statistical significance was determined by one-way analysis of variance followed by Dunnett’s post-hoc test, two-tailed Student’s t test or Mann-Whitney test. (*p < 0.05, **p < 0.01, ***p < 0.001 versus the Con or Normal group).

To investigate the mechanism of bile acid–induced cytotoxicity on trophoblasts during ICP, we treated HTR8 cells and human primary trophoblasts (HPT) from term placentas with CA/DCA/CDCA and then tested cell viability and mitochondrial integrity. As hydrophobic bile acids at very high concentration would cause cellular necrosis, we did not include 200 μM bile acids in the further assays. Treatment with 50 and 100 μM hydrophobic bile acids (DCA, CDCA) showed dose-dependent effects on deleterious cell viability, increased ROS production and reduced mitochondrial membrane potential (ΔΨm) in HTR8 and HPT cells (Fig. 3A–C and Supplementary Fig. S1A–C). Whereas treatment with a less hydrophobic bile acid^20^, CA (100 μM), moderately affected cell viability, ROS production and mitochondrial activity in HTR8 cells (Fig. 3A–C). In addition, 100 μM DCA and CDCA treatment reduced cellular adenosine triphosphate (ATP) content in HTR8 cells and in villous explants (Fig. 3D and Supplementary Fig. S1D). Furthermore, by quantitative reverse transcription polymerase chain reaction (qRT-PCR) assay, we demonstrated that DCA and CDCA (100 μM) significantly decreased the levels of mitochondrial gene transcripts (*MT-CO1, MT-ND1* and *MT-ND6*) (Fig. 3E) and mitochondrial DNA (mtDNA) copy number (Fig. 3F). A similar change was also observed in placentas from ICP patients (Fig. 3G,H).

### Knockdown of PRDX3-induced oxidative stress and mitochondrial dysfunction in HTR8 cells

**Figure 4:**
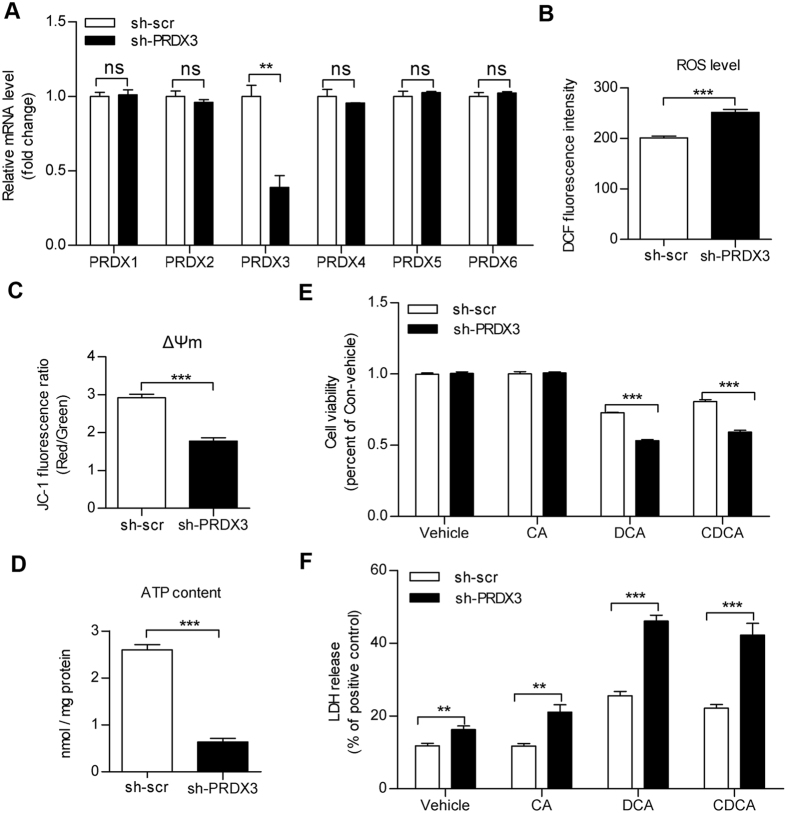
Knockdown of peroxiredoxin-3 (PRDX3)-induced oxidative stress and mitochondrial dysfunction. HTR8 cells were transiently transfected with PRDX3 shRNA plasmid (sh-PRDX3) or scramble shRNA (sh-scr). (**A**) The messenger RNA expressions of PRDX family members (PRDX1-6) were determined by qRT-PCR with normalisation to 18s rRNA level. (**B**) Reactive oxygen species production, (**C**) mitochondrial membrane potential (ΔΨm) and (**D**) adenosine triphosphate concentration were assayed at 36 hours post-transfection. Results for reactive oxygen species and mitochondrial membrane potential were expressed as arbitrary units, whereas adenosine triphosphate (ATP) level was expressed as nmol/mg protein. (**E**,**F**) Twenty-four hours after transfection, HTR8 cells were treated with 100 μM cholic acid, deoxycholic acid and chenodeoxycholic acid for 24 hours. (**E**) Cell viability assay and (**F**) quantification of lactate dehydrogenase release were applied. The results were expressed as percentage of control. Data were shown as the mean ± standard error of mean. Statistical significance was determined by two-tailed Student’s t test. (**p < 0.01, ***p < 0.001 versus the sh-scr group).

Our previous study^8^ and aforementioned results in this study suggested that PRDX3 might be involved in toxic bile acids–induced trophoblastic damage, which was associated with the oxidative stress and mitochondrial dysfunction. To further investigate the role of PRDX3 in trophoblast cells, we transfected HTR8 cells with shPRDX3 or scramble shRNA plasmids. As shown, compared with other members of PRDX family, the level of PRDX3 mRNA was specifically knocked down by PRDX3 shRNA transfection (Fig. 4A). As expected, knockdown of PRDX3 significantly induced mitochondrial dysfunction, as indicated by overproduction of ROS, loss of mitochondrial membrane potential and decline in ATP content (Fig. 4B–D). Furthermore, PRDX3 knockdown reduced viability and promoted lactate dehydrogenase (LDH) release in DCA/CDCA-treated cells (Fig. 4E,F).

### Knockdown of PRDX3 induced growth arrest in trophoblast cells

**Figure 5:**
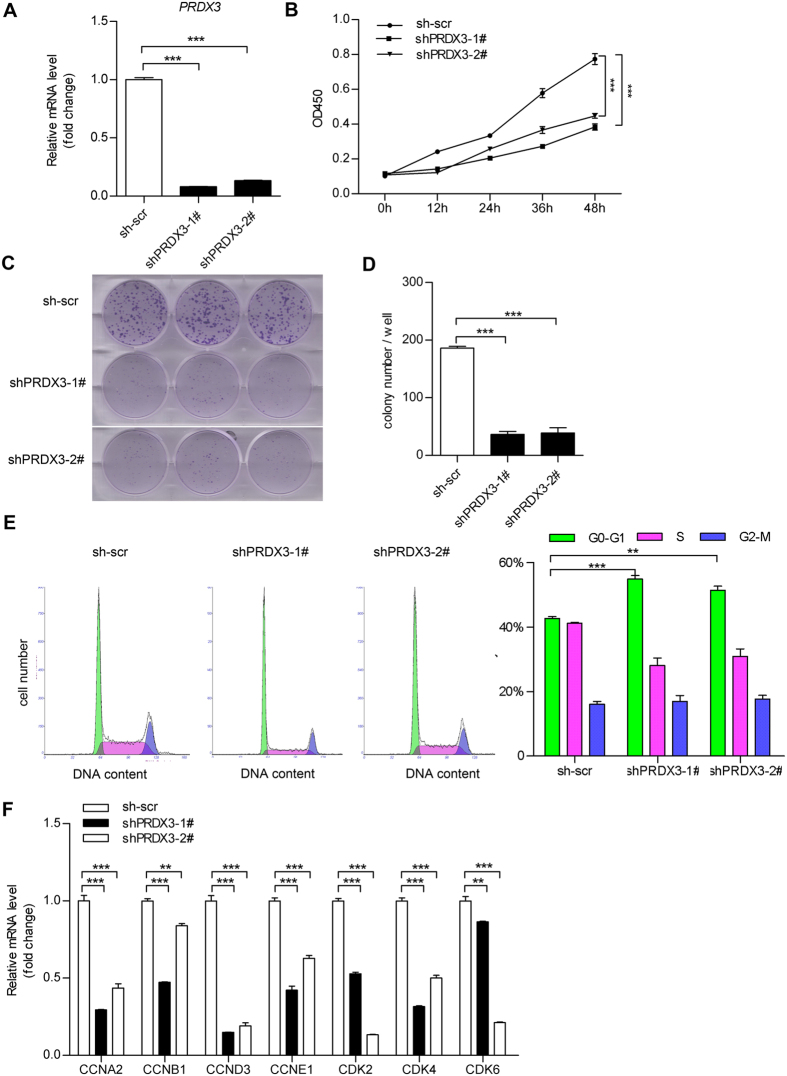
Knockdown of peroxiredoxin-3 (PRDX3) expression induced trophoblast cell growth arrest. HTR8 cells were stably transfected with PRDX3 shRNA plasmid (sh-PRDX3) or scramble shRNA (sh-scr). One stable clone (sh-scr) for control and two clones for stable PRDX3 knockdown (shPRDX3-1# and shPRDX3-2#) were selected and expanded. (**A**) Stable transfected cell lines were subjected to qRT-PCR assay to detect PRDX3 level. (**B**) Cell proliferation assay for the stably transfected cells were applied at the indicated times (n = 6, ***p < 0.001 versus the sh-scr group, two-way analysis of variance). (**C**) Growth rates of the stable transfected cells were also determined using the colony formation assay as described in “Materials and Methods”. (**D**) Colony numbers stained by crystal violet were quantified from (**C**). (**E**) Stably transfected cells were harvested and stained with PI/RNase solution and subjected to cell cycle analysis by flow cytometry. (**F**) Total RNA from the stably transfected cell lines were subjected to qRT-PCR assay for determination of messenger RNA levels of cell cycle progression genes (including *Cyclin A2, Cyclin B1, Cyclin D3, Cyclin E1, CDK2, CDK4* and *CDK6*). Data were shown as mean ± standard error of mean. Statistical significance was determined by two-way analysis of variance or one-way analysis of variance followed by Dunnett’s post-hoc test. (**p < 0.01, ***p < 0.001 versus the sh-scr group).

To further analyse the effect of PRDX3 in trophoblasts, we developed stably transfected HTR8 cell strains with plasmids expressing short hairpin RNA (shRNA) against PRDX3 (shPRDX3) or control scramble shRNA. HTR8 cell clones expressing sh-PRDX3 (shPRDX3–1# and shPRDX3-2#) and scrambled control (sh-scr) were selected and expanded (Fig. 5A). In addition, we observed that the mtDNA copy number and the levels of mitochondrial gene transcripts were apparently decreased after PRDX3 knockdown (Supplementary Fig. S2A,B).

To clarify the effect of PRDX3 on growth of trophoblast cells, the PRDX3-knockdown clones (shPRDX3-1# and shPRDX3-2#) or the control clone (sh-scr) were applied for CCK-8 cell proliferation assay and colony formation assay. The growth rates (Fig. 5B) and the numbers of colony formation of PRDX3-knockdown cells were much lower than the control clone (Fig. 5C,D). Subsequently, the cell cycle distributions of these cell strains were analysed with flow cytometer. Compared to the sh-scr cells, shPRDX3-1# and shPRDX3-2# cells exhibited prolonged G0/G1 phase (Fig. 5E), indicative of growth arrest. Furthermore, cell cycle regulation–related genes were determined. As shown in Fig. 5F, the mRNA levels of cell cycle progression genes (including *Cyclin A2, Cyclin B1, Cyclin D3, Cyclin E1, CDK2, CDK4* and *CDK6*) were significantly reduced in PRDX3-knockdown cells compared with scrambled control cells.

In addition, we also observed a decreased number of cytotrophoblastic cells in placentas from ICP patients compared with placentas from normal pregnancies, as evidenced by lower proportion of p63 positive immunostaining (Supplementary Fig. S3A,B), which is a marker for cytotrophoblasts in placenta^21^,^22^.

### Decreased PRDX3 expression promoted cellular senescence through p38 activation and p16/p21 induction

**Figure 6:**
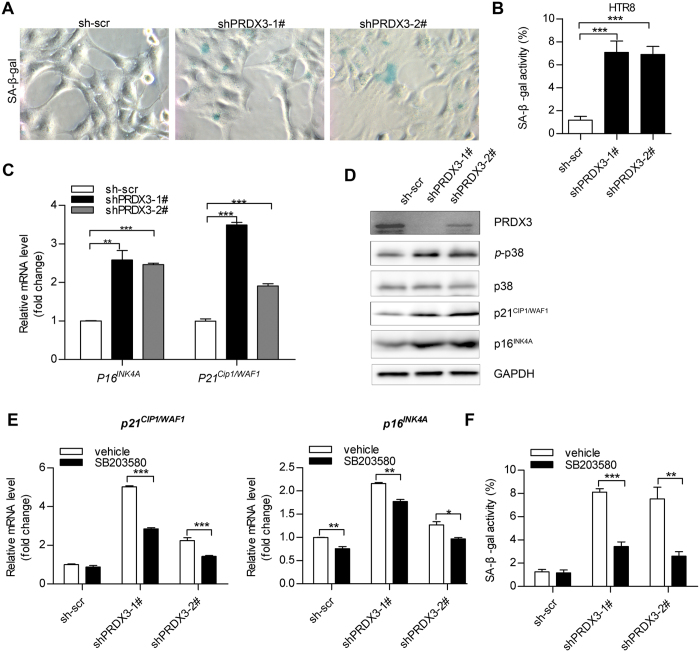
Decreased peroxiredoxin-3 (PRDX3) expression promoted cellular senescence through inducing p38 activation and p21/p16 expression. (**A**) HTR8 clones expressing sh-PRDX3 (shPRDX3-1# and shPRDX3-2#) or scrambled control (sh-scr) were stained for DAPI and SA-β-Gal as described in “Materials and Methods”. Representative images were shown (original magnification 400×). (**B**) The total cell number was determined by nuclear fluorescence signal (DAPI). Quantification of SA-β-gal staining positive cells relative to total cells was shown. The value represents the mean ± standard error of mean of three randomly selected fields. (**C**) QRT-PCR was performed for the analysis of p21^WAF1/CIP^ and p16^INK4A^ messenger RNA levels in HTR8 cells stable clones. (**D**) Immunoblot assay for detecting p38-mitogen-activated protein kinase activation (p-p38), p21^WAF1/CIP^ and p16^INK4A^ protein level was applied. Full-length blots were presented in Supplementary Fig.S9. (**E**,**F**) HTR8 stable clones (sh-scr, shPRDX3-1# and shPRDX3-2) were treated with p38-mitogen-activated protein kinase inhibitor SB203580 (10 μM) or vehicle (DMSO) for 24 hours. Cells were subjected to analysis of p21^WAF1/CIP^ and p16^INK4A^ messenger RNA levels by qRT-PCR assay (**E**) or quantification of senescent cells number by SA-β-gal staining (**F**). Data were shown as mean ± standard error of mean. Statistical significance was determined by two-tailed Student’s t test or one-way analysis of variance followed by Dunnett’s post-hoc test. (*p < 0.05, **p < 0.01, ***p < 0.001 versus the sh-scr group).

Accumulation of oxidative stress and cell-cycle arrest would result in cellular senescence^23^,^24^. The increased activity of senescence-associated β-galactosidase (SA-β-gal) is used as a biochemical marker for cellular senescence^24^. In our study, PRDX3-knockdown cells showed a significantly higher number of SA-β-gal stained cells compared with sh-scr control cells at basal level (Fig. 6A,B). p21^WAF1/CIP^ (also known as CDKN1A) and p16^INK4A^ (also known as CDKN2A) were two well-established senescence-associated molecular markers^25^,^26^, which were upregulated in senescent cells^22^,^23^. qRT-PCR assay revealed that mRNA levels of p21^WAF1/CIP^ and p16^INK4A^ were increased in PRDX3-knockdown cells compared with sh-scr cells (Fig. 6C). Immunoblot assay also confirmed the corresponding change of protein levels of p21^WAF1/CIP^ and p16^INK4A^ in PRDX3-knockdown cells (Fig. 6D), and in ICP placentas by immunoblot and IHC assays (Supplementary Figs S4 and S5). The oxidative stress–induced p38-MAPK activation was also involved in cellular senescence^27^,^28^. Indeed, we observed elevated p38-MAPK phosphorylation in PRDX3-knockdown cells (Fig. 6D). Inhibition of p38-MAPK by specific inhibitor, SB203580 reduced p21^WAF1/CIP^ and p16^INK4A^ mRNA level (Fig. 6E) and blunted cellular senescence in PRDX3-knockdown cells (Fig. 6F). Taken together, these data demonstrated that stable knockdown of PRDX3K in HTR8 cells promoted cellular senescence via activating p38-MAPK and increasing p21^WAF1/CIP^ and p16^INK4A^ expression.

## Discussion

The placenta plays a key role in transportation of bile acids at the foetal-maternal interface and protection of the foetus against hydrophobic bile acids–induced cytotoxicity in both human and rodent^29^,^30^. Excessive accumulation of toxic bile acid–induced oxidative stress is a severe insult to placenta during the development of ICP^13^. In rat hepatocytes, DCA and CDCA promoted ROS generation and cell death^31^. The accumulated hydrophobic bile acids in the placental compartment of a rat OCP model resulted in substantial oxidative stress–induced cellular damage and apoptosis^9^. Moreover, the placental antioxidant system was impaired instead of being augmented in this model, indicating that the feedback regulation was disrupted under OCP^9^. Consistent with these findings, we also reported that enhanced oxidative stress in mouse placentas accompanied with impaired antioxidant system in maternal cholestasis model^8^. In the present study, we demonstrated that the expression of the mitochondrial antioxidant protein PRDX3 was decreased in ICP placentas and toxic bile acids–treated trophoblastic cells and villous explant. On one side, toxic bile acids induced trophoblast oxidative stress by increasing intracellular ROS level. On the other side, reduced PRDX3 level by toxic bile acids led to impairment of antioxidant system in trophoblasts. As a result, the homeostasis of oxidation-antioxidation in trophoblasts was interrupted by accumulation of bile acids in placenta during the progression of ICP.

As evidenced by hypomethylated PRDX3 promoter in placentas and unaffected PRDX3 level upon 5-Aza-2′-deoxycytidine treatment in trophoblasts, we proposed that DNA methylation was not responsible for PRDX3 downregulation in ICP placenta. However, bile acids treatment indeed reduced PRDX3 levels in trophoblast cells. The nuclear factor erythroid 2-related factor 2 (NFE2L2/NRF2), which functioned as a predominant transcription factor for intracellular antioxidant response, was reported to be an upstream regulator of PRDX3^32^,^33^. We also found that knockdown of NRF2 by siRNA led to PRDX3 downregulation in HTR8 cells (Supplementary Fig. S6A). Moreover, the mRNA level of *NRF2* was significantly reduced in ICP placentas and hydrophobic bile acids (DCA and CDCA)–treated trophoblastic cell line (Supplementary Fig. S6B,C). Our data suggest that bile acids induced downregulation of NRF2 may be one of the causes of decreased PRDX3 expression in ICP. However, the underlying mechanism requires further investigation.

Bile acids–induced mitochondrial dysfunction might be involved in the pathogenesis of ICP. Severe intracellular oedema was commonly observed in villous trophoblast cells in placentas from ICP patients and mouse model of maternal cholestasis^8^, which might be associated with mitochondrial dysfunction in trophoblast. We showed here that treatment by medium to high levels of hydrophobic bile acids caused oxidative stress and mitochondrial dysfunction (reduced mitochondrial membrane potential and ATP concentration) in trophoblasts. Similar results were also observed in toxic bile acids–treated hepatic cell lines^34^,^35^.

Production and accumulation of ROS in mitochondria is the dominant source of intracellular oxidative stress. ROS could modify lipids, mitochondrial DNA and proteins, resulting in mitochondrial membrane oxidative damage and mitochondrial structure impairment^36^. PRDX3 plays an important role in maintaining mitochondrial oxidation-reduction homeostasis. Weakened mitochondrial antioxidant defences by bile acids treatment or PRDX3 knockdown with shRNA resulted in reduced mitochondrial membrane potential and ATP content. The mitochondrial respiratory chain complexes may be damaged by the deregulated ROS^37^, which further resulted in reduced mitochondrial membrane potential. The electrochemical gradient of protons across the inner membrane of mitochondria would also be dissipated due to decreased ATP production^38^. Our observations were consistent with a previous report that reduced levels of another mitochondrial antioxidant, MnSOD, also resulted in oxidative stress and mitochondrial dysfunction in mouse liver^39^.

Less cytotrophoblasts indicated by p63 and higher percentage of p21^WAF1/CIP^ and p16^INK4A^ staining were observed in ICP placenta. These results indicated bile acids-promoted oxidative stress would induce trophoblastic growth arrest in placenta. Moreover, our results supported the involvement of PRDX3 in proliferation control of trophoblast. We demonstrated that knockdown of PRDX3 induced growth arrest of trophoblast cells. The role of PRDX3 in regulating cell growth has been widely studied in cancer cells^37^,^40^. In colon cancer, PRDX3 was overexpressed in colon cancer stem cells and essential for maintenance of and survival of colon cancer stem cells^41^. Chua *et al*. also reported that siRNA mediated silencing of PRDX3 inhibited breast cancer cells proliferation and induced cell cycle arrest^42^. However, it was also demonstrated that overexpression of PRDX3 in thymoma cells resulted in decreased cell proliferation. Thus, the function of PRDX3 in cell growth regulation was dependent on cellular background^43^. Accumulation of oxidative stress and cell cycle arrest would result in cellular senescence^23^,^24^. Until now, the role of PRDX3 in placental trophoblast cells senescence has not been reported. We found that the percent of SA-β-gal–positive staining cells and the cellular senescence markers p21^WAF1/CIP^ and p16^INK4A^ were obviously increased in trophoblast cells with PRDX3 stably knocked down. Thus, silencing of PRDX3 would promote oxidative stress–induced cellular senescence.

In summary, the present study demonstrated that the expression of PRDX3 was reduced in ICP placentas and in hydrophobic bile acids–treated trophoblasts and villous explant. Silencing of PRDX3 in trophoblasts promotes oxidative stress and mitochondrial dysfunction. Moreover, PRDX3 knockdown induced cell growth arrest and cellular senescence via activation of p38 MAPK and induction of p21^WAF1/CIP^ and p16^INK4A^. Thus, decreased expression of PRDX3 in ICP placenta may serve as an alternative mechanism for toxic bile acid–induced placenta injury and antioxidant dysfunction. Our results further highlight the role of PRDX3 in protecting trophoblast against toxic bile acids–induced oxidative damage during ICP.

## Methods

### Clinical Samples

Term placentas were collected within 30 minutes after birth through vaginal delivery or caesarean section. Full-thickness samples were taken from within the central two-thirds of the placenta disc. Three specimens in 0.5 cm × 0.5 cm areas for cryo-storage (−80 °C) and three specimens in 2 cm × 1 cm areas for formalin fixed and paraffin embedded (FFPE) were taken from one placenta. For preparing cryo - tissue, the placenta tissue was washed with PBS, snap-frozen in liquid nitrogen and stored at −80 °C until use. For preparing FFPE tissue, the placenta tissue was fixed with 10% neutral-buffered formalin at 4 °C for at least 48 hours, following dehydration with gradient ethanol and paraffin embedding. Frozen tissues (12 from women with normal pregnancy and 12 from women with ICP) and paraffin embedded tissue blocks (46 from women with normal pregnancy and 70 from women with ICP, Table 1) of human term placentas were retrieved from the archives of Departments of Pathology and Bio-bank, the International Peace Maternity and Child Health Hospital, affiliated to Shanghai Jiao Tong University School of Medicine. To diagnose ICP, the commonly accepted clinical criteria were applied^3^,^5^,^44^. Inclusion criteria were maternal serum TBA ≥ 10 μmol/L and presence of classical pruritus during gestation without primary skin diseases, or raised serum liver transaminases level in some cases. Exclusion criteria were presence of preeclampsia, gestational diabetes *mellitus*, HELLP syndrome (haemolysis, elevated liver enzymes and low platelets), viral hepatitis, primary biliary cholangitis, acute fatty liver of pregnancy and foetal abnormality. Fasting serum bile acids concentration in the ICP patients of this study was shown in Supplementary Table S1. Ethical approval for the use of tissues in this study was obtained from the institutional ethical review board of the International Peace Maternity and Child Health Hospital, Shanghai Jiao Tong University School of Medicine. The written informed consents were obtained from all participants for the use of placental samples during non-commercial medical investigations. All experiments involving human samples were performed in accordance with relevant institutional and national guidelines and regulations.

### Immunohistochemistry Staining

Immunohistochemistry (IHC) assays for detecting protein level of PRDX3, p21^CIP1/WAF1^, p16^INK4A^, and p63 in human placenta tissues were applied using standard protocol as previously described^45^. Three blocks from each placenta were assayed for each protein. Briefly, placental 5 μm thick sections were deparaffinised and boiled in 1 mM ethylenediaminetetraacetic acid buffer for 15 minutes for antigen retrieval. Primary antibodies were was applied and incubated overnight at 4 °C. Normal rabbit immunoglobulin G (IgG) was used as the negative control. Subsequently, the sections were incubated with the *SuperPicture* IHC Detection Kit Horseradish Peroxidase Polymer (Invitrogen, Carlsbad, CA, United States) and then developed by 0.05% diaminobenzidine. Mayer’s haematoxylin was used as a counterstain.

The sections of IHC staining were evaluated using Leica DM2500 microscope (Leica Microsystems, Wetzlar, Germany) at 200X magnification. The images were visualised by LAS V4.6 software (Leica Microsystems) in a computer. At least five random fields per section were chosen. Protein expressions in syncytiotrophoblasts and cytotrophoblasts from terminal villi were assessed. The sections were evaluated by two pathologists (ZHJ & LY) separately. Discrepancies were discussed until a consensus was reached. For PRDX3, which is ubiquitously expressed in trophoblast plasma, the intensity of the immunostained signal was evaluated as 1+ (weak staining covers more than 50% of the terminal villi), 2+ (moderate staining covers more than 50% of the terminal villi) or 3+ (strong staining covers more than 50% of the terminal villi). For p21^CIP1/WAF1^ and p16^INK4A^, the positive stained nuclei were counted. The positive nuclei were counted in about 1000 nuclei. The expression of p21^CIP1/WAF1^ and p16^INK4A^ were graded according to the scores: 0 (less than 1% of positive cells), 1 (1–10% of positive cells), 2 (10–20% of positive cells), 3 (more than 20% of positive cells). For p63, the positive stained nuclei were counted in at least 1000 trophoblastic cells from 10 random fields/section. The percentage of positive labelled nuclei/total number of nuclei counted was calculated as previously reported^21^.

### Protein Extraction and Immunoblot Assay

Placental tissues were lysed in ice-cold RIPA lysis buffer (150 mM NaCl, 1% NP-40, 0.5% deoxycholate, 0.1% SDS, 50 mM Tris–HCl) by homogenisation. Cell lysate was prepared in a SDS lysis buffer (Beyotime Institute of Biotechnology, Jiangsu, China). Protease and phosphatase inhibitors (Biotool LLC, Houston, TX, United States) were added in the lysis buffers. Immunoblot assays were applied as previously reported^45^. In brief, total protein lysate was separated by SDS-PAGE in 10% or 12% gels and then transferred to a polyvinylidene difluoride membrane (Millipore, Billerica, MA, United States). Primary antibodies and secondary antibodies used are listed in Supplementary Table S2. Subsequently, the membrane was developed by using enhanced chemiluminescent assay kit (Tiangen Biotech, Beijing, China) under ImageQuant LAS 4000 mini imager (GE Healthcare, Piscataway, NJ, United States). Densitometric analysis was performed using Image Quant TL software (GE Healthcare).

### Quantitative PCR and qRT-PCR Assay

Total DNA was extracted from cells and tissues using a commercial kit (Tiangen, Beijing, China). Total RNA was isolated from cells and tissues using Trizol reagent (Invitrogen, Carlsbad, CA, United States). RNA extraction was applied following the standard protocol employing chloroform-isopropanol-ethanol steps for purification. Isolated RNA samples were quantified using NanoDrop 2000c spectrophotometer (Thermofisher Scientific, Wilmington, DE, United States). To eliminate genomic DNA contamination, RNA samples were treated with AccuRT gDNA Removal Kit (ABMgood Inc., Richmond BC, Canada) before reverse transcription. Subsequently, cDNA was synthesised using EasyScript Reverse Transcriptase (ABMgood) according to the manufacturer’s instruction. Quantitative PCR for detecting mRNA level and mtDNA copy number was performed using a miScript SYBR Green PCR kit (Qiagen, Hilden, Germany) on a StepOnePlus Real-time PCR system (Applied Biosystems, Foster City, CA, United States). Primers for corresponding genes in quantitative PCR and qRT-PCR assays were listed in Supplementary Table S3. Relative quantification of mtDNA copy number and mRNA levels was applied by using the 2^−∆∆Ct^ method with normalisation to the endogenous control as previously described^8^.

### Methylation-specific Quantitative PCR

DNA methylation analysis was applied using *EpiJET* DNA Methylation Analysis Kit (Thermo Fisher Scientific, Waltham, MA, United States) as previously described^46^,^47^. The kit contains two isoschizomers *HpaII* and *MspI*, both of which recognise 5′-C^CGG-3′ sites. However, *HpaII* cannot cleave DNA if CpG within its restriction sites is methylated, whereas *MspI* digests the sequence neglect of methylation status. Briefly, DNA sample was digested by each individual enzyme. Undigested DNA (no enzyme added as a control) and the DNA after digestion were quantified using quantitative PCR with a pair of primers (5′-CTTCTCAGACAGGACCCCG-3′ and 5′-GTTACCCGCGGAAACCCTC-3′) that flank PRDX3 promoter region. The percentage of methylated DNA was calculated according to the manufacturer’s instruction, by comparing the remaining amount of DNA in digestion reaction with that of undigested control.

### Villous Explant Culture

Villous explant culture from the term placenta was performed according to previous reports^48^,^49^. In brief, fresh human term placental tissues were collected from healthy women with pregnancy (37–39 gestational weeks) The villi were collected and dissected into small pieces. After several cycles of dissection and washing, the explant fragments were incubated in suspension in DMEM/F12 medium (Hyclone, Logan, UT, United States) supplemented with 10% fetal bovine serum (Gibco, Rockville, MD, United States) and penicillin, streptomycin and amphotericin B solution (Gibco) at 37 °C, 5% CO_2_, 8% O_2_ in a multi-gas incubator (MCO-5M, Panasonic Corporation, Osaka, Japan).

### Cell Culture and Transfection

Human primary trophoblasts from term placentas were isolated by digestion with 0.25% trypsin and 300 U/ml deoxyribonuclease-I (Sigma-Aldrich, St. Louis, MO) for 3 cycles of 20 minutes at 37 °C according to a previously described protocol^50^. The obtained cells were pooled and purified using discontinuous Percoll (5–70%, GE healthcare) density gradient centrifugation. Furthermore, differential adhesion separation in cell culture plates was also applied to remove the easily attached fibroblasts and suspended blood cells^45^. Primary trophoblasts were cultured in DMEM/F12 medium supplemented with 10% fetal bovine serum and 1% antibiotics (penicillin, streptomycin, and amphotericin B) at 37 °C, 5% CO_2_. Cells were treated with bile acids after overnight culture.

Human choriocarcinoma cell line JAR was obtained from Shanghai Cell Bank of Chinese Academy of Sciences (Shanghai, China) and maintained in Dulbecco’s modified Eagle’s medium (Invitrogen) supplemented with 10% foetal bovine serum (Invitrogen) and 1% penicillin/streptomycin in a Thermo Scientific incubator (37 °C, 5% CO_2_). The immortalised trophoblast cell line HTR8/SVneo (HTR8), was established previously^51^. HTR8 cells were cultured in RPMI 1640 supplemented with 10% foetal bovine serum and 1% penicillin/streptomycin.

PRDX3 or scramble shRNA plasmids were purchased from Genechem co., Ltd (Shanghai, China). Target sequences for PRDX3 shRNA and scrambled control are 5′-TAAGCCTTGATGACTTTAA-3′ and 5′-TTCTCCGAACGTGTCACGT-3′, respectively. The constructs were confirmed by DNA sequencing. Transient transfection with shRNA plasmids was applied using lipofectamine 2000 (Invitrogen) according to the manufacturer’s instructions. For selection of stable silenced clones, transfected HTR8 cells were incubated in RPMI-1640 containing 2 μg/ml puromycin (Sigma-Aldrich). After 3 weeks of selection, individual colonies were isolated and expanded. The expression of PRDX3 in these clones was confirmed by qRT-PCR and immunoblot analysis. The stably transfected lines were maintained with 1 μg/ml puromycin.

### Cell Viability and Cell Proliferation Assays

Cell viability and cell proliferation assays were conducted using the Cell Counting Kit-8 (CCK-8) from Dojindo (Kamimashiki-gun, Kumamoto, Japan) according to the manufacturer’s instruction. Briefly, HTR8 cells were seeded on 96-well plates at a density of 1 × 10^4^ cells/well and maintained at 37 °C under 5% CO_2_. At each time point, cells were incubated with CCK-8 reagent for 1 hour at 37 °C, followed by measuring the optical absorbance at 450 nm with the SpectraMax M3 microplate reader (Molecular Devices, Sunnyvale, CA, United States). All experiments were carried out in triplicate.

### Mitochondrial Membrane Potential Measurement

Mitochondrial membrane potential (ΔΨ_*m*_) was assayed using the JC-1 fluorescence dye (Shanghai Yeasen Biotechnology Co., Shanghai, China) as previously described^35^. In brief, 1 × 10^4^ adherent cells in 96-well black plates were incubated with culture medium containing 2 μg/ml JC-1 at 37 °C for 15 minutes in dark. Fluorescent intensities for the green signal (JC-1 monomer, Ex = 485 nm, Em = 530 nm) and red signal (JC-1 aggregate, Ex = 525 nm, Em = 590  nm) were measured respectively using the SpectraMax M3 microplate reader (Molecular Devices). The mitochondrial membrane potential was determined by calculating the ratio of red to green fluorescence intensity. All experiments were carried out in triplicate.

### Intracellular ROS Level Determination

Intracellular ROS of HTR8 cells was determined using a ROS Assay Kit (Shanghai Yeasen Biotechnology Co.), which contains an oxidation sensitive fluorescent dye 2,7-dichlorofluorescin diacetate, as described elsewhere^34^,^35^. Briefly, adherent cells in 96-well black plates were incubated with 5 μM dichlorofluorescin diacetate in serum-free culture medium at 37 °C for 30 minutes in dark. After three washings with phosphate buffered saline, intracellular ROS was analysed by detecting the dichlorodihydrofluorescein fluorescence intensity with Ex = 480 nm and Em = 525 nm under the SpectraMax M3 microplate reader (Molecular Devices). Arbitrary units were used to express the fluorescent intensity. All experiments were carried out in triplicate.

### LDH Cytotoxicity Assay

The percentage of LDH released from injured cells was determined using the LDH Cytotoxicity Assay Kit (Beyotime Institute of Biotechnology). In brief, HTR8 cells cultured in 96-well plates were treated with corresponding bile acids for 24 hours. The activities of LDH in culture medium and cell extracts were measured according to the manufacturer’s protocols. The optical absorbance at 490 nm was recorded with the SpectraMax M3 microplate reader (Molecular Devices). All experiments were carried out in triplicate.

### Measurement of Cellular ATP Levels

Intracellular ATP content measurement was applied using a luminescence-based ATP assay kit (Beyotime Institute of Biotechnology) according to the manufacturer’s instructions. Briefly, HTR8 cells or villous explant were resuspended in a lysis buffer from the kit. After centrifugation, the supernatant was mixed with the ATP detection solution. The intensity of luminescence was recorded in a SpectraMax M3 microplate reader (Molecular Devices). ATP concentrations were determined by using a standard curve and expressed as nmol/mg protein.

### Cell Cycle Analysis

Cells were collected by trypsin digestion, and fixed in ice-cold 70% ethanol at 4 °C overnight. After several washing steps, cells were stained with propidium iodide/RNase staining solution (BD Biosciences, Rockville, MD, United States) for 30 minutes at 37 °C. DNA content was quantified using Beckman Coulter flow cytometer with standard optics (Beckman Coulter, Miami, FL, United States). The Multicycle Program (Beckman Coulter) was used to calculate the percentage of cells in G0-G1/S/G2-M phases of the cell cycle as described previously^45^.

### Colony Formation Assays

Cell cultures were harvested with trypsin-ethylenediaminetetraacetic acid (Invitrogen, Carlsbad, CA, United States) and counted using Cellometer-1000 cell counter (Nexcelom Bioscience, Lawrence, MA, United States). Cells were diluted in complete medium, and 1,000 cells were plated in a 6-well tissue culture plate. After incubation for 12 days at 37 °C under 5% CO_2_, the cells were washed with phosphate buffered saline, fixed with paraformaldehyde (4%) and stained with 0.5% Crystal Violet for 30 minutes at room temperature. Plates were washed in tap water three times and dried overnight. The size and density of visible colonies were evaluated. Colonies with more than 25 cells were counted.

### Senescence-Associated β-Galactosidase Assay

Senescence-associated β-galactosidase (SA-β-gal) activity was assessed using cellular senescence assay kit from Beyotime Institute of Biotechnology according to the manufacturer’s instructions. Briefly, stably transfected HTR8 cells were fixed and stained with X-gal at pH = 6.0. Positive stained cells with clear blue cytoplasmic signal were counted. In addition, nuclei of cells were stained by DAPI (Beyotime Institute of Biotechnology). The number of total cells was determined by nuclear fluorescent signals. Photos were taken in three randomly chosen fields per dish using a Leica DM2500 microscope (Leica Microsystems). For quantification, the percentage of SA-β-gal–positive stained cells relative to total cells was determined.

### Statistics

Data were shown as means ± standard error of mean. Statistical analysis was performed using the GraphPad Prism 5 software (GraphPad Software, San Diego, CA, United States) and Statistical Package for Social Science 16.0 (SPSS Inc., Chicago, IL, United States) by two-tailed Student’s t test, Mann-Whitney test, one-way analysis of variance (ANOVA) followed by Dunnett’s post-hoc test, Fisher’s exact test, chi-square test or two-way analysis of variance. A value of *p* < 0.05 was considered statistically significant.

## Additional Information

**How to cite this article**: Wu, W.-B. *et al*. Downregulation of peroxiredoxin-3 by hydrophobic bile acid induces mitochondrial dysfunction and cellular senescence in human trophoblasts. *Sci. Rep.* **6**, 38946; doi: 10.1038/srep38946 (2016).

**Publisher’s note:** Springer Nature remains neutral with regard to jurisdictional claims in published maps and institutional affiliations.

## Supplementary Material

Supplementary Material

## Figures and Tables

**Table 1:** Clinical characteristics of the subjects.

	Normal (n = 46)	ICP (n = 70)	*p* value
Maternal age (years)	30.5 ± 3.2	30.2 ± 4.0	0.648
BMI of mother	21.3 ± 2.5	21.8 ± 3.0	0.359
Gravidity	1.7 (1–4)	1.7 (1–7)	0.473
Parity	1.3 (1–2)	1.2 (1–3)	0.063
Gestational age (weeks)	38.9 ± 1.0	37.6 ± 1.6	<0.0001
TBA at delivery (μmol/L)	5.8 ± 2.9	29.4 ± 24.1	<0.0001
Birth weight (g)	3368.6 ± 354.6	3056.7 ± 395.1	<0.0001
Placenta weight (g)	538.1 ± 77.4	488.0 ± 91.7	<0.01
Apgar at 5 min	9.98 (9–10)	9.94 (7–10)	0.545
Sex of neonate			
Male (%)	18 (39.1)	39 (55.7)	0.081
Female (%)	28 (60.9)	31 (44.3)	

Abbreviation: ICP, intrahepatic cholestasis of pregnancy; BMI, body mass index; TBA, total bile acids. Values are given as mean ± SD, mean (range), or N (percent).
