# Supplementary material for: Downregulation of peroxiredoxin-3 by hydrophobic bile acid induces mitochondrial dysfunction and cellular senescence in human trophoblasts
Source: Sci Rep. 2016 Dec 13;6:38946. doi: 10.1038/srep38946 (PMC5154184; doi:10.1038/srep38946)
Supplement: Supplementary Material [file srep38946-s1.pdf]

Supplementary information

Downregulation of peroxiredoxin-3 by hydrophobic bile acid induces mitochondrial dysfunction and cellular senescence in human trophoblasts

Wei-Bin Wu [a,b], Ramkumar Menon [c], Yue-Ying Xu [a], Jiu-Ru Zhao [a,b], Yan-Lin Wang [d] , Yuan Liu [a], Hui-Juan Zhang [a,b,*]

[a] Departments of Pathology and Bio-Bank, the International Peace Maternity and Child Health Hospital , Shanghai Jiao Tong University School of Medicine, Shanghai, China;

[b] Institute of Embryo-Fetal Original Adult Disease Shanghai Key laboratory for Reproductive Medicine, School of Medicine, Shanghai Jiao Tong University, Shanghai, China;

[c] Division of Maternal-Fetal Medicine and Perinatal Research, Department of Obstetrics and Gynecology, the University of Texas Medical Branch at Galveston, Galveston, Texas, United States of America;

[d] Prenatal Diagnosis Center & Fetal Medicine Unit, the International Peace Maternity and Child Health Hospital, Shanghai Jiao Tong University School of Medicine, Shanghai, China.

*To whom correspondence should be addressed. Tel: 86-021-64070434-29905. Fax: 86-021-64073896. Email: zhanghj815@126.com.

Supplementary table S1. Serum bile acids concentration in the ICP patients of this study.

Patient No.	TBA level at delivery (μM)	TBA peak during pregnancy (μM)
1	14.6	14.6
2	18.6	18.6
3	47.9	47.9
4	10.2	70
5	15.7	15.7
6	22.1	29.9
7	38.8	40.2
8	126.2	126.2
9	13.8	24.5
10	93.2	93.2
11	99.8	99.8
12	39.1	39.1
13	6.8	37.7
14	42.1	93.4
15	8.7	37.4
16	18.4	18.4
17	14.7	61.7
18	51.2	51.2
19	48.4	48.4
20	42.9	42.9
21	37.9	61.6
22	20.9	20.9
23	16.2	16.2
24	18.4	18.4
25	15.4	15.4
26	39.7	39.7
27	15.3	101.8
28	15.1	25.2
29	24.2	38
30	15.4	97.9
31	15.3	44.6
32	24.6	48.9
33	27.4	27.4
34	22	27.2
35	28.8	116.2
36	28.7	28.7
37	18.6	21.6
38	14.2	19.5

39	15.1	15.1
40	18.8	18.8
41	59	59
42	24.9	24.9
43	15.6	15.6
44	14.6	40.2
45	16.8	22.8
46	15.6	32.9
47	15.8	15.8
48	27.8	27.8
49	24	24
50	20.1	20.1
51	18.8	18.8
52	32.1	32.1
53	134.4	134.4
54	23.2	23.2
55	33.6	45.3
56	24.3	79.7
57	30.7	30.7
58	17.9	17.9
59	20.8	23.8
60	29.2	29.2
61	16.6	63.9
62	52.4	52.4
63	21.9	21.9
64	16.4	16.4
65	45.4	45.4
66	18.4	18.4
67	31.9	31.9
68	14.4	33.7
69	23.5	37.1
70	15.9	39.3

Supplementary table S2. Antibodies used for immunoblot assay (IB) and immunohistochemistry (IHC).

Antibodies	Companies	Dilution
Rabbit anti-PRDX3	Abgent, Suzhou, China	IB(1:1500)<br>IHC (1:100)
Rabbit anti-β-Actin	Abmart Inc., Shanghai, China	IB(1:5000)
Goat anti- $P16^{INK4A}$	R&D Systems, Minneapolis, MN, United States	IB(1:1000)
Rabbit anti-$P16^{INK4A}$	Proteintech Group Inc., Chicago, IL, United States	IHC (1:300)
Rabbit anti-p63	Cell Signaling Technology, Danvers, MA, United States	IHC (1:100)
Rabbit anti-$P21^{WAF1/CIP1}$	Cell Signaling Technology, Danvers, MA, United States	IB (1:1000)<br>IHC (1:50)
Rabbit anti-phospho-p38 (Thr180/Tyr182) MAPK	Cell Signaling Technology, Danvers, MA, United States	IB(1:1000)
Rabbit anti- p38 MAPK	Cell Signaling Technology, Danvers, MA, United States	IB(1:1000)
Mouse anti-GAPDH	Abmart Inc., Shanghai, China	IB(1:5000)
HRP-conjugated Goat anti-rabbit IgG	Jackson ImmunoResearch,West Grove, PA, United States	IB(1:2500)
HRP-conjugated	Jackson ImmunoResearch,West Grove, PA, United	IB(1:2000)

Goat anti-mouse IgG	States	
HRP-conjugated Rabbti anti-goat IgG	Jackson ImmunoResearch, West Grove, PA, United States	IB(1:2000)

Supplementary table S3. Primers for qPCR assay

Gene	Alias	Forward Primer	Reverse Primer
For RT-qPCR assay			
PRDX1	PRX1	TCTCCAAGCAGAAGTGAGCG	GAAAGGCTGGTCTCTCCACC
PRDX2	PRX2	GTCCAGGCCTTCCAGTACAC	CTGTCATCCACGTTGGGCTT
PRDX3	prx-III	ACAGCCGTTGTCAATGGAGAG	ACGTCGTGAAATTCGTTAGCTT
PRDX4	PRX-4	AGAGGAGTGCCACTTCTACG	GGAAATCTTCGCTTTGCTTAGGT
PRDX5	prx-V	CTTCACCCCTGGATGTTCCAA	AGGCATCATTAACACTCAGACAG
PRDX6	PRX	GACTCATGGGGCATTCTCTTC	CAAGCTCCCGATTCCTATCATC
RNA18S5	*18S rRNA*	CAGCCACCCGAGATTGAGCA	TAGTAGCGACGGGCGGTGTG
MT-CO1	COX1	ACTATACTACTACTAACAGACCG	GGTTCTTTTTTTCCGGAGTA
MT-ND1	ND1	TAGTCTCAGGCTTTAACATCG	AGTTGGTCGTAGCGGAAT
MT-ND6	ND6	GGGGAATGATGGTTGTCT	TCATACTCTTTCACCCACAG
CCNA2	Cyclin A2	CGCTGGCGGTACTGAAGTC	GAGGAACGGTGACATGCTCAT
CCNB1	Cyclin B1	TTGGGGACATTGGTAACAAAGTC	ATAGGCTCAGGCGAAAGTTTTT
CCND3	Cyclin D3	AACTTGGCTGAGCAGAGCAC	CATCCGAACAGAGCCAGTCT
CCNE1	Cyclin E1	ACTCAACGTGCAAGCCTCG	GCTCAAGAAAGTGCTGATCCC
CDK2		GAGCCTGGGCTGCATCTTTG	CCCACTTGGGGAAACTTGGC
CDK4		ATGGCTACCTCTCGATATGAGC	CATTGGGGACTCTCACACTCT
CDK6		TGCACAGTGTCACGAACAGA	AGATCGCGATGCACTACTCG
CDKN2A	$p16^{INK4A}$	ACTTCAGGGGTGCCACATTC	CGACCCTGTCCCTCAAATCC
CDKN1A	$p21^{WAF1/CIP1}$	GGATGTCCGTCAGAACCC	GCTCCCAGGCGAAGTCA
For qPCR assay			
mtDNA		CCCTAAAACCCGCCACATCT	GAGCGATGGTGAGAGCTAAGGT

gDNA		CAACTTCATCCACGTTCACC	GAAGAGCCAAGGACAGGTAC

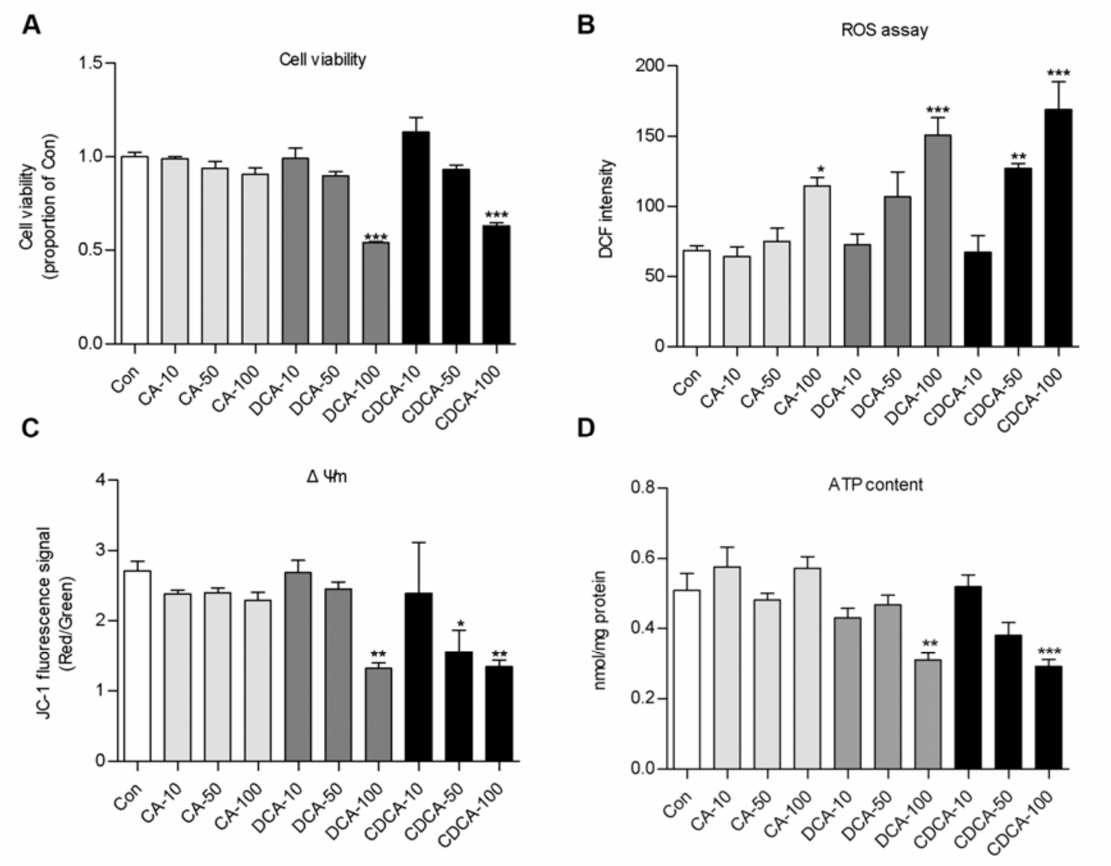

Supplementary Fig. S1. Primary trophoblasts cells (A–C) and villous explants from term placenta (D) were treated with vehicle (Con), 10, 50, or 100 μM cholic acid (CA), deoxycholic acid (DCA) and chenodeoxycholic acid (CDCA) for 24 hours. (A) Cell viability, (B) ROS production, (C) mitochondrial membrane potential (ΔΨm) and (D) adenosine triphosphate (ATP) content were assayed using corresponding kits. The ATP level was shown as nmol/mg protein and the others were expressed as arbitrary units. (*$p<0.05$, **$p<0.01$, ***$p<0.001$ versus the Con group)

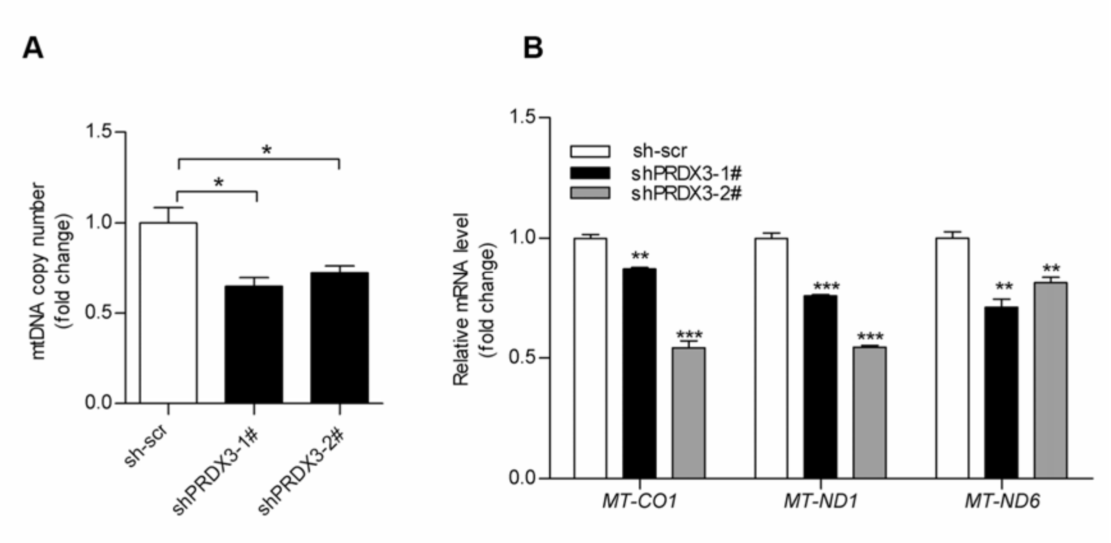

Supplementary Fig. S2. qRT-PCR and qPCR analysis was applied to detect (A) mitochondrial DNA (mtDNA) copy number and (B) mitochondrial gene transcripts (MT-CO1, MT-ND1 and MT-ND6) levels in PRDX3-knockdown cells (shPRDX3-1# and shPRDX3-2#) or the control cells (sh-scr). (*$p<0.05$, **$p<0.01$, ***$p<0.001$ versus the sh-scr group)

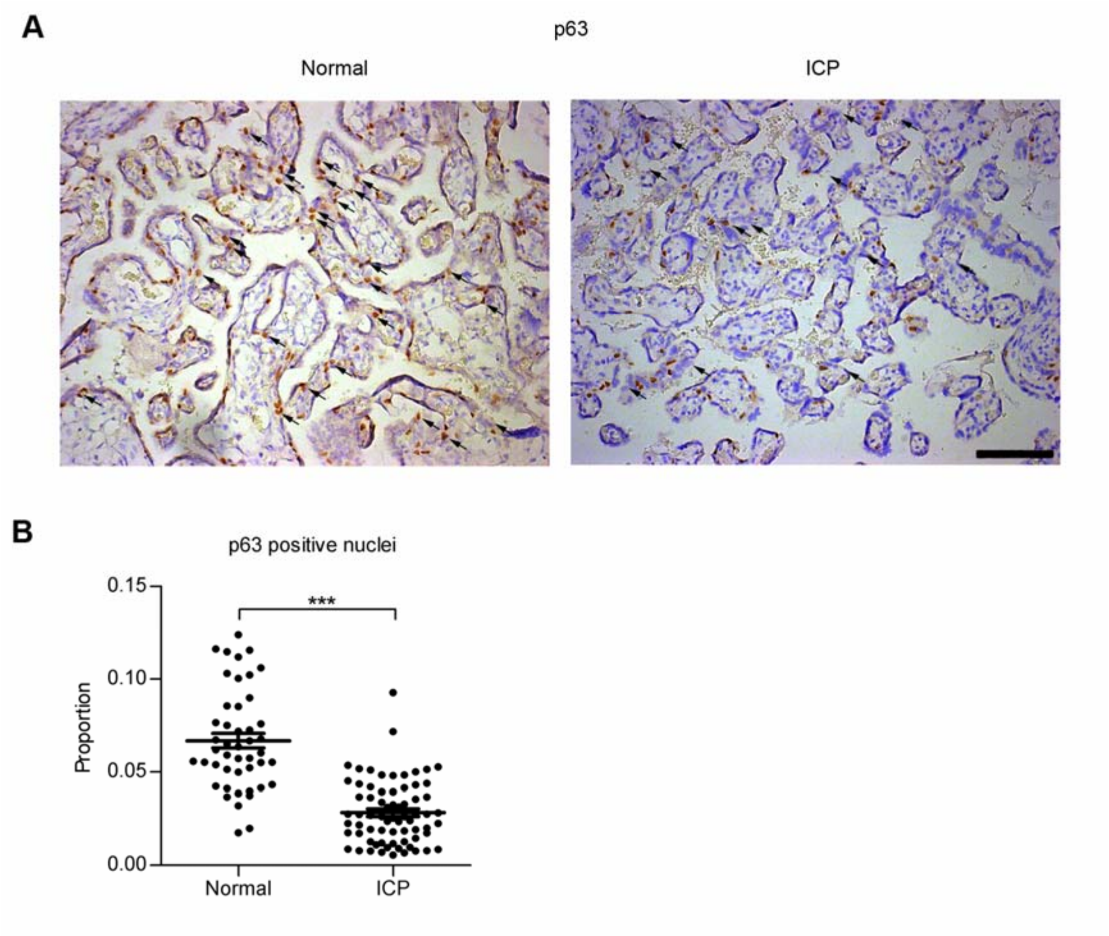

Supplementary Fig. S3. (A) Immunohistochemistry staining of p63 for cytotrophoblasts in term placentas from ICP patients and normal pregnancies. Original magnification 200 x, bar: 100 μm. (B) The proportion of positive labelled nuclei / total number of trophoblastic nuclei counted was determined in a minimum of 1000 trophoblasts from 10 random fields / section. Statistically analysis of immunostaining of p63 in placentas was applied (n = 46 for normal pregnancy, n = 70 for ICP, ***p <0.001, Mann-Whitney test).

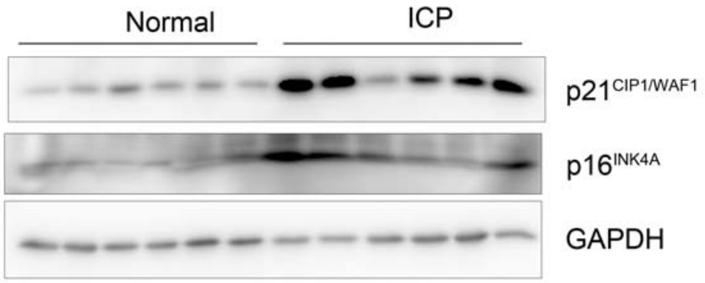

Supplementary Fig. S4. Immunoblot assay for detecting $p21^{WAF1/CIP}$ and $p16^{INK4A}$ protein level in placental tissues from normal pregnancies and ICP patients. GAPDH was used as an internal loading control.

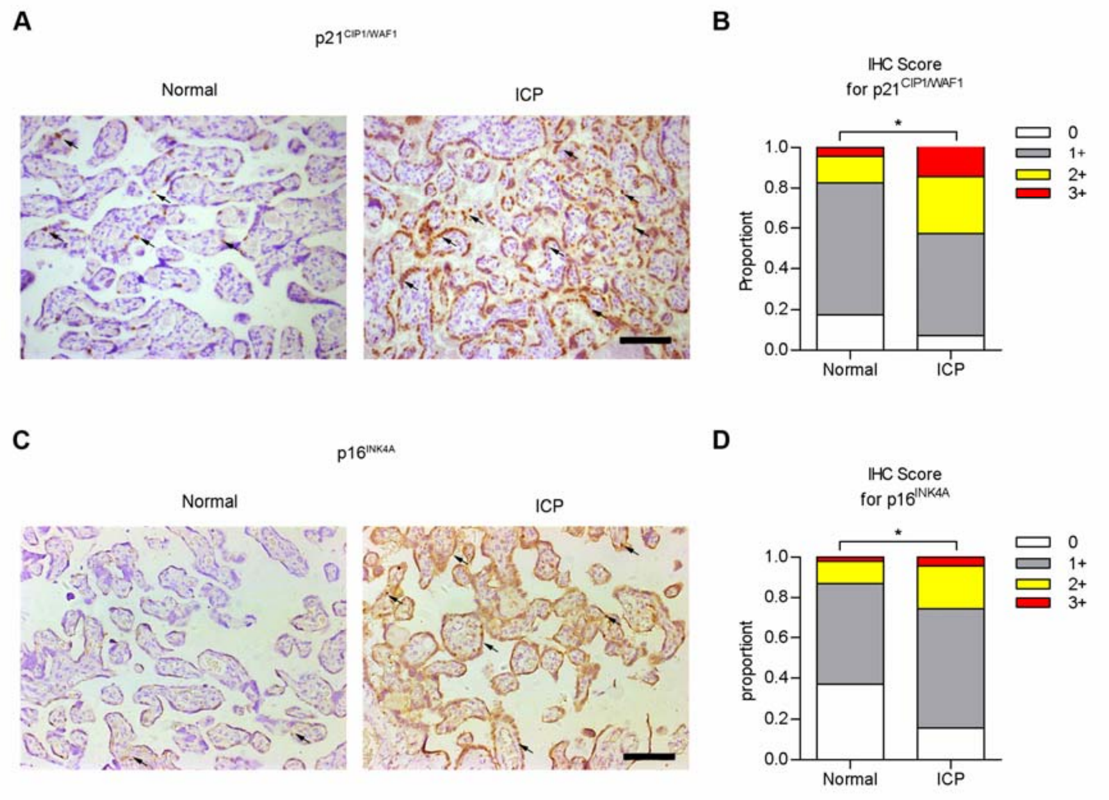

Supplementary Fig. S5. Immunohistochemistry analysis and score of $p21^{CIP1/WAF1}$ (A, B) and $p16^{INK4A}$ (C, D) in trophoblasts of placental tissues. The expressions of $p21^{CIP1/WAF1}$ and $p16^{INK4A}$ were scored as: 0 (less than 1% of positive cells), 1+ (1-10% of positive cells), 2+ (10-20% of positive cells), 3+ (more than 20% of positive cells). Original magnification 200x, bar 100 μm. Statistically analysis of immunostaining signal of $p21^{CIP1/WAF1}$ (B) and $p16^{INK4A}$ (D) in placentas (n = 46 for normal, n = 70 for ICP, *p <0.05, chi-square test).

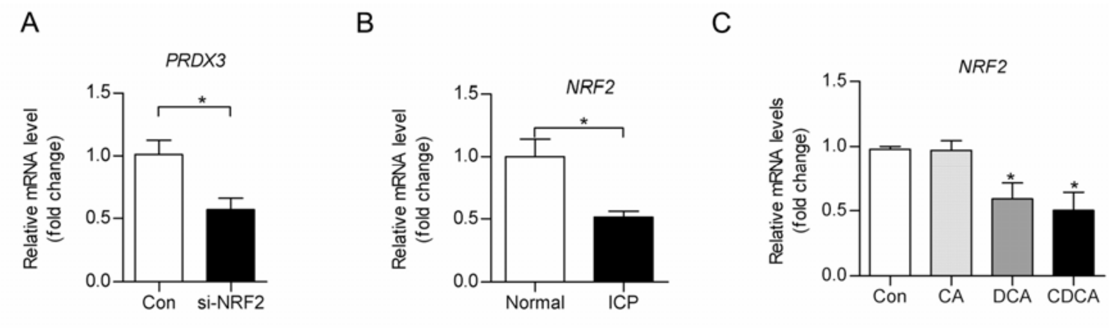

Supplementary Fig. S6. (A) mRNA level of PRDX3 in HTR8-SVneo cells transfected with NRF2 siRNA, (B) mRNA level of NRF2 in placentas from normal pregnancies or ICP patients and (C) mRNA level of NRF2 in HTR8-SVneo cells treated with 100 μM CA, DCA or CDCA was quantified by qRT-PCR assay with normalization to 18s rRNA level. (*$p<0.05$ versus the Con or Normal group)

For Fig.1A

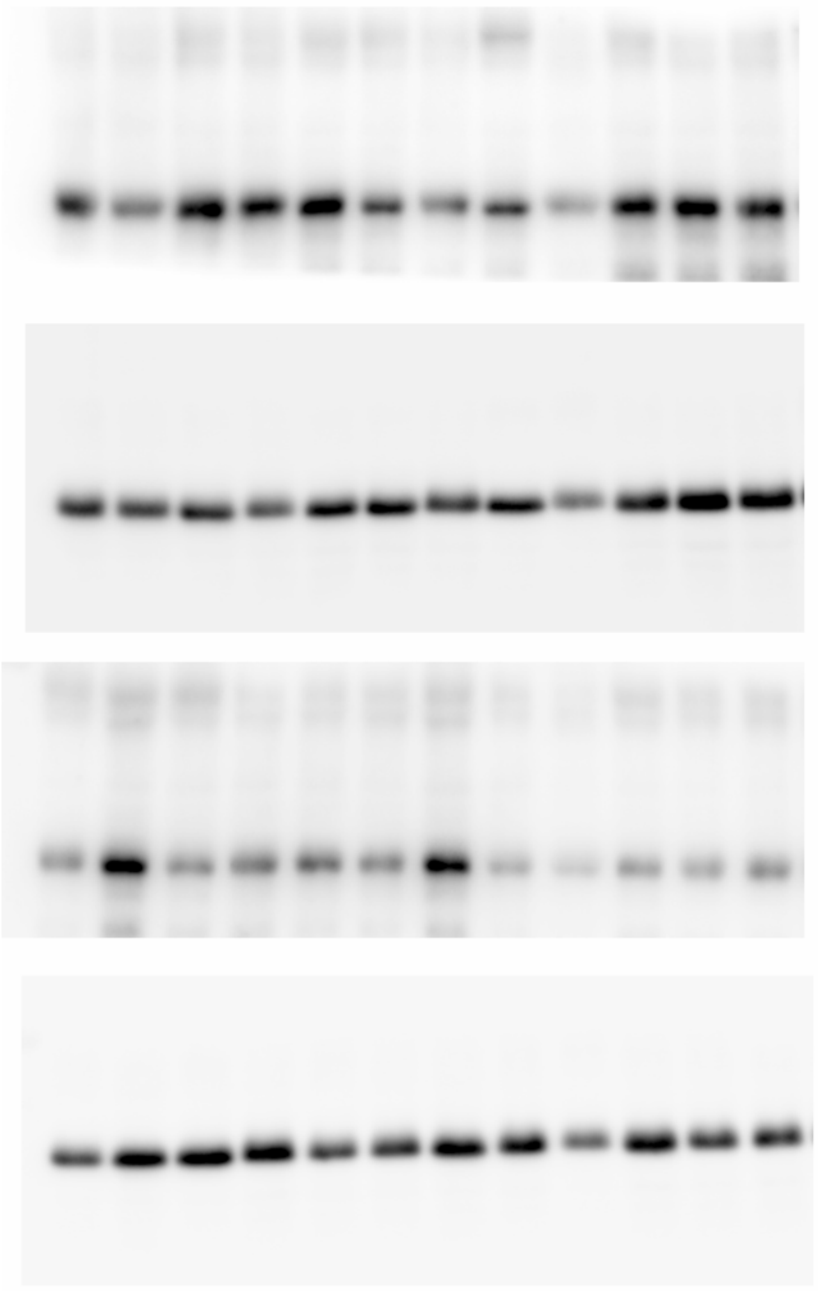

Supplementary Fig.S7. Full-length blots for Fig.1A.

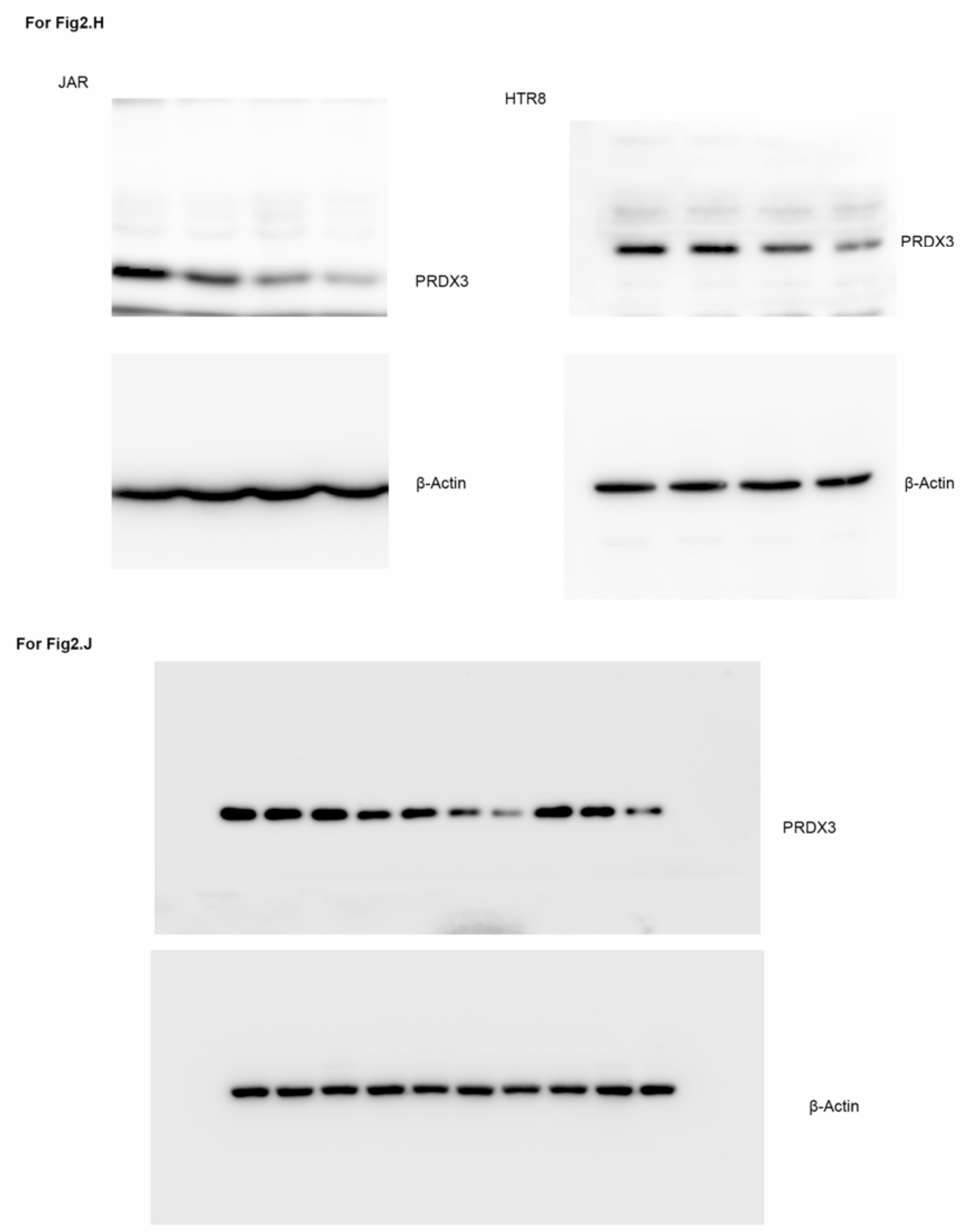

Supplementary Fig.S8. Full-length blots for Fig.2H, J.

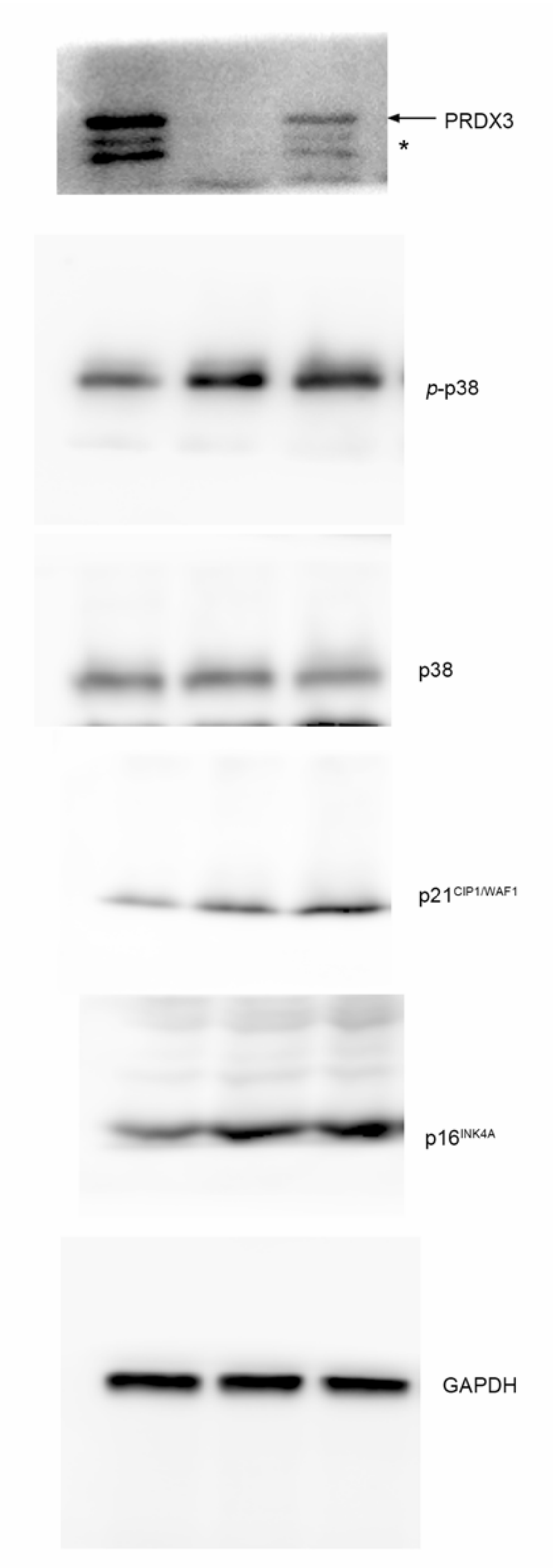

Supplementary Fig.S9. Full-length blots for Fig.6D. * non-specific bands
